# Co-designing a participatory evaluation of older adult partner engagement in the mcmaster collaborative for health and aging

**DOI:** 10.1186/s40900-024-00595-x

**Published:** 2024-06-11

**Authors:** Marfy Abousifein, A. Tina Falbo, Joyce Luyckx, Julia Abelson, Rebecca Ganann, Brenda Vrkljan, Soo Chan Carusone

**Affiliations:** 1 McMaster Collaborative for Health and Aging, McMaster University, Hamilton, Canada; 2 School of Nursing, McMaster University, Hamilton, Canada; 3 Department of Health Research Methods, Evidence and Impact, McMaster University, Hamilton, Canada; 4 School of Rehabilitation Science, McMaster University, Hamilton, Canada

**Keywords:** Partnership, Equity, Patient and public involvement

## Abstract

Engagement of patients and the public in health research is crucial for ensuring research relevance and alignment with community needs. However, there is a lack of nuanced evaluations and examples that promote collaborative and reflective learning about partnerships with partners. The aim of this paper is to provide a case example of a participatory evaluation of the engagement of older adult partners in an aging-focused research centre. We outline our process of co-planning and implementing an evaluation of the McMaster Collaborative for Health and Aging’s engagement strategy through the use of multiple methods, including a standardized tool and qualitative approaches. The team chose to explore and capture the engagement experiences and perspectives of the older adult partners within the Collaborative using a survey (the Public and Patient Engagement Evaluation Tool (PPEET)), an art-based method (photovoice), and a focus group. We present a brief summary of the findings but primarily focus this paper on the experiences of using each methodology and tool, with an emphasis on promoting dialogue on the benefits, limitations, and challenges. We reflect on the process of co-planning and the integration of both standardized tools and qualitative approaches to adopt a holistic approach to evaluating partnership within the Collaborative. Ultimately, this case example aims to provide practical guidance for other research groups navigating the complexities of partnership engagement and evaluation, thereby promoting meaningful partnerships in research.

**Supplementary Information:**

The online version contains supplementary material available at 10.1186/s40900-024-00595-x.

## Abstract (plain language summary)

Engaging older adults in research is important to match research study goals with older adult needs and interests and can lead to better health outcomes and a more equitable healthcare system. Yet, older adults are often excluded from research due to the idea that they can be a challenging group with whom to work. The McMaster Collaborative for Health and Aging is a provincial research centre established to engage patients, researchers, and other key partners to improve the health of and the healthcare system for older adults. The Collaborative partners with older adults and caregivers in all its activities and projects. For example, older adults mentor students and advise on patient-partnered research projects and co-facilitate workshops. There are many frameworks and models to support research partnerships with people with lived experience. Yet, evaluating the quality and impacts of these partnerships is less common. This paper presents a case example of a participatory evaluation of older adult partners’ engagement in the Collaborative. Together, older adult partners and researchers decided on the project goals and methods. The evaluation used multiple methods to capture partners’ experiences and perspectives on the process and impact of their engagement in research. First, a survey called the Public and Patient Engagement Evaluation Tool (PPEET) was completed. Second, photovoice was used (a method involving photography for self-expression). Finally, a focus group was conducted (discussions with partners facilitated by researchers). We reflect on the benefits and limitations of each method and make recommendations for future evaluations. The paper underscores considering partner preferences and abilities when choosing evaluation methods for patient-partnered research.

**Supplementary Information:**

The online version contains supplementary material available at 10.1186/s40900-024-00595-x.

## Introduction

Patient, caregiver, and public involvement in health and social care research can ensure that research goals and priorities are relevant and align with the goals, interests, and needs of both patients and the public [1, 2]. Identifying patient-defined priorities has been shown to improve health outcomes and promote a “sustainable, accessible, and equitable healthcare system” [3]. Moreover, if the research population in question is marginalized or equity- denied, such as older adults, many argue there is a moral obligation that such research be conducted in collaboration with this population [3]. In Canada and other Western countries, individuals aged 65 + represent the fastest-growing segment of the population and the largest age group of users of the healthcare system [4]. Healthcare expenditure per person for the 80–85 age group in 2017 was more than double the average across all age groups in Canada [5]. To transform the health system to meet the needs of the aging population and support person-centred care, older adults should be engaged as partners in health research. Despite this need, older adults are engaged in research less often than other groups due to the perception that they are vulnerable or hard to engage as patient partners [6].

The Canadian Institutes of Health Research (CIHR) indicates in its Strategy for Patient-Oriented Research (SPOR) that patients should be engaged in ‘active and meaningful collaboration’ as partners in the research process [2]. A diverse array of frameworks, models, and best practices support SPOR’s objectives, including a recent focus on evaluating these partnerships [2, 7, 8]. Examples from the literature include (1) discussions of validated tools assessing patient engagement, such as the Patient Engagement in Research Scale (PEIRS) [2], (2) frameworks for guiding the integration of patient partners [7], and (3) a review of the literature regarding themes within these guiding frameworks [8]. While such literature builds a foundation for researchers to understand what is important when engaging partners and evaluating them, it lacks the detail and examples to guide researchers on how to do so in a good way and learn from the mistakes of others who have.

To support this paradigm shift in research and ensure that research is being done through authentic partnerships, where risks and harms are minimized, we must not just change how we do research but also evaluate these changes and respond to these findings. Tools and guidance exist to evaluate partnerships, such as those published by the United Kingdom’s National Co-ordinating Centre for Public Engagement (NCCPE) [9]. NCCPE promotes creative evaluation method exploration through their “Evaluation Inspiration Board” where researchers can share their experience with different evaluation methods [9]. However, published case examples to demonstrate application of such tools and reflections on their use are limited [9, 10]. Reed at al.propose a common standard for evaluating partnership based on NCCPE’s tools, however, the proposal does not focus on evaluations of partnerships that are done in partnership and was not developed in partnership [10]. Consequently, there is a focus on the researcher-identified goals when assessing partner engagement in research. While there is an increasing number of evaluation papers published that draw on quantitative data and involving patient partners, there is still a lack of case examples about evaluating partnerships [2, 7, 8]. More nuanced evaluations regarding the impact of partnerships and examples of evaluations that empower a reflective approach to partnerships are needed to promote learning from experiences. By bringing a participatory approach to the evaluation of patient partner engagement, we hope to demonstrate how this practice might become more embedded in future participatory projects ultimately promoting meaningful partnerships from both the researcher and partner perspective. There is a prevailing saturation of reports on evaluation outcomes, and a relative dearth of discussion on methodology and considerations for process. Addressing this gap, this paper focuses on the methodology and process of an evaluation rather than evaluation results [11, 12].

## The McMaster collaborative for health and aging

The Ontario SPOR SUPPORT Unit (OSSU) is a network of 14 leading health research centres located in the province of Ontario, Canada that encompasses eight research initiatives that engage researchers, patients and other partners in patient-oriented research to improve the health of Ontarians and the health care system [13]. OSSU provides infrastructure, expertise and support to people engaged in patient-oriented research, promotes knowledge transfer and exchange of the latest research evidence, and aims to improve health policy and clinical practices in Ontario [13].

As one of the research centres in the OSSU network, the McMaster Collaborative for Health and Aging (herein, the Collaborative) aims to improve the health and well-being of older Canadians by advancing patient-oriented health research on aging [13, 14]. The aim of the Collaborative, is to optimize the health and longevity of the aging population as well as transform the experience of aging by upholding transparency and collaboration [14–17]. The Collaborative manifests such goals by collaborating with older adults in all activities [15]. Examples of these activities include (1) supporting and advancing patient-oriented research with a focus on advancing health equity and optimizing aging, (2) mobilizing knowledge to improve healthcare systems and practices to ultimately improve the health and well-being of older Canadians, and (3) supporting patient-oriented research capacity building and partnerships. The Collaborative’s mandate is to provide capacity building and support patient-oriented research on aging [15].

Within the Collaborative, a group of nine older adult partners (women = 5, men = 4), aged 60–80, meet monthly via Zoom with the managing director. In 2023, each partner had been part of the Collaborative for a minimum of 6–12 months. Partners have diverse health and health care experiences. The group includes individuals living with multiple chronic diseases and those with disabilities requiring mobility aids, such as motorized wheelchairs, as well as those with lived experiences as caregivers to older adults living with physical and/or cognitive decline. The team advises and supports the Collaborative’s operations and initiatives, including workshop planning and mentorship of graduate students on engagement strategies. Depending on their interests and experience, these partners also assume more specialized roles, such as serving on the graduate funding review committee or Leadership Council or co-presenting at workshops. Partners receive individual support to facilitate meaningful participation at a level and depth that works for them and to mitigate barriers due to education or health status. Partners receive an honorarium for their contributions ($25 CAD/hour).

Conventional evaluations of partnerships are typically from the researchers’ perspectives and employ traditional methods like focus groups. The Collaborative presents an opportunity to evaluate the involvement and perspectives of older adult partners in such initiatives and roles while facilitating introspection and learning from the partnership. To improve our own work and strengthen our working partnerships, we conducted a participatory evaluation of older adult engagement with the Collaborative using multiple methods.

## Objective

This paper provides a case example of a participatory evaluation of the engagement of older adult partners in an aging-focused SPOR research centre, the McMaster Collaborative for Health and Aging. We outline our process of co-planning and implementing an evaluation of the Collaborative’s engagement strategy through the use of multiple methods, including a standardized tool and qualitative approaches.

## Positionality statement

In the spirit of self-reflexivity, MA acknowledges her standpoint as a Canadian immigrant and health sciences undergraduate student researcher who led the participatory evaluation of the engagement of older adult partners in the Collaborative. Prior to this project, MA was not involved in the engagement of the partners in the Collaborative thereby, reducing the potential for bias and power dynamics within the process of evaluation. While this means that MA has not established a relationship with the partners, the supervisor (SCC) and other research team members have been the main point of contact for the partners throughout their time with the Collaborative. As a result, members of the research team were familiar with the older adults, which provided a foundation of trust, allowing for constructive critiques and power-sharing. MA facilitated all activities described in this paper.

## Funding

Funding for the student lead was provided by a McMaster Institute for Research on Aging Undergraduate Summer Research Fellowship.

## Co-designing the evaluation

The full team of older adult partners (*n* = 9) were invited to participate in this participatory evaluation project. All nine were interested but two partners required a short leave from the Collaborative, due to health and personal reasons, during the planning and data collection phases of this study. The team of researchers, including older adult partners, the student lead, and research team, identified the overarching goals of the project. The primary goal was to evaluate the engagement and experience of older adults in the Collaborative, during each individual’s involvement, to identify what they identified as successes from their perspective and to identify ways to improve partnership approaches going forward. We aimed to use the outputs of the evaluation to inform and promote meaningful improvements within the Collaborative’s operations and partnerships.

A participatory approach was chosen to evaluate partnership as studies show that participatory evaluation advances a research team or organization’s capacity to meaningfully engage diverse stakeholders [18]. In participatory approaches, techniques sensitive and responsive to community needs are used to engage stakeholders and ensure a team/organization’s approaches are as effective as possible [18]. Additionally, working in partnership to develop the evaluation aligns with the values and goals of the Collaborative. Power redistribution is a crucial benefit of participatory approaches considering that partners have decision making power in the design and thus impact of evaluations. Research shows that participatory evaluations lead to more ownership and institutional buy-in and thus increase the likelihood that outcomes of the results will be used to improve partnerships by promoting accountability [19–21].

In May 2023, as a first step to planning the evaluation, MA and SCC invited all nine older adult partners to a 90-minute meeting. Seven partners attended and two absences were due to health and personal reasons. The objective of this meeting was to decide on the evaluation methods. To come to this decision, we discussed the distinction and strengths and limitations of qualitative and quantitative research methods, and the partners were presented with different options that have been used in evaluation studies (including standardized surveys, focus groups, poetry-based data collection, and photovoice). Each option’s description, advantages, and disadvantages were presented in accessible language. Then, as a team, we discussed the options, and the partners were encouraged to ask questions and offer suggestions for other methods with the aim of choosing the methods most appropriate for our team and the study objectives. The partners wanted an evaluation plan that would provide a holistic portrayal of their experiences and allow for multiple methods of self-expression, increasing evaluation accessibility. This perspective has been supported in the literature as the use of both quantitative and qualitative methods bring different strengths to the research and can improve the depth and breadth of the research outcomes [22, 23].

As a group, we decided to use the Public and Patient Engagement Evaluation Tool (PPEET) [24]. This tool has been specifically designed for qualitative and quantitative analysis (see evaluation methods below). Partners identified that capturing experiences qualitatively was valuable to provide individuals with the opportunity to vocalize thoughts and perspectives that cannot be captured with numbers, ultimately leading to a more holistic evaluation. They wanted to capture these qualitative aspects through more than one mode of data collection and emphasized the benefits of including personal reflections as well as reflections based on discussion with the whole partner group. We presented them with the pros and cons of focus groups, and art-based methods, such as poetry and photovoice activities. The group was interested in an arts-based approach as well as a more traditional focus group. These methodological decisions were reached through discussion followed by consensus with 100% agreement by all members. To increase dialogue, meeting facilitators often invited and encouraged quieter partners to express their perspectives and welcomed diverse and contradictory opinions.

For planning the focus group and arts-based methods, a voting system was used to decide on the interview questions (see “focus group” section for questions) as well as the type of arts-based method we would use; these voting data were gathered online through Microsoft Forms. For those who needed help accessing and using the forms, they were provided with alternative options such as voting via a phone call/email or provided individual support with using the form. Focus group questions were developed, in partnership with the Collaborative partners, to address aspects of the Collaborative partners’ experiences which they felt were not sufficiently captured by the PPEET alone. Partners wanted additional opportunities to provide unrestricted feedback as well as the opportunity to discuss successes and limitations of partnership as a group. Additionally, the partners agreed on the use of photovoice as their arts-based method to answer the following prompt: “What would the ideal partnership/engagement of older adults be in the Collaborative?” The photovoice method was chosen by the partners, as they appreciated the individual and reflective elements of the method [25, 26]. They liked that the method provided them time alone with the prompt and allowed expression of their ideas in non-verbal ways. This method was appropriate for the prompt since it inquired about idealistic expectations of partnership, which partners shared required time and reflection to determine the answer for.

These methodological decisions were largely led by what was perceived as the best way to address the evaluation objectives.

## The evaluation methods

### Public and patient engagement evaluation tool (PPEET) [24]

The PPEET survey is available in multiple languages, such as English, French, and Dutch. The survey was created in 2011 as a product of a Canadian collaboration at McMaster University composed of researchers and public and patient engagement practitioners. Many benefits of the PPEET lie in its continuous improvement, as a result of surveying diverse stakeholders who implemented the tool and shared their experiences of success and challenges. This feedback led to the latest version of the PPEET published in 2018. Due to its wide usage, there is a rich literature validating the tool for different cultures and settings thereby improving its applicability to assessing partnerships in range of circumstances [27–30].

For this project, we used the participant questionnaire version 2.0 from the PPEET, which assesses various features of engagement, including: (1) the organization’s capacity for meaningfully engaging diverse stakeholders and culture of public and patient engagement, (2) participants’ assessments of their engagement, and (3) the planning, execution and impact of the engagement activity after it has been completed. The participant questionnaire is designed to obtain participants’ assessments of features of an engagement initiative. This 22-question survey includes sections on communication and support for participation, sharing views and perspectives, and impacts and influence of the engagement initiative. Respondents are asked to indicate their level of agreement, from strongly disagree to strongly agree, with statements such as “I am able to express my views freely.” The questionnaire included additional open-ended questions. The partners had the opportunity to complete this questionnaire from July to September of 2023. Individual support over the phone was provided to mitigate impairments or barriers with technology use. The PPEET allowed for anonymous expression of perspectives, which is important to allow partners to mention negative aspects in their experience. A total of seven partners participated.

### Photovoice

Photovoice serves as a qualitative method employed to tackle intricate and sensitive subjects, providing individuals with a platform to candidly express their viewpoints [25, 26]. By employing photographs taken and chosen by the participants, individuals are able to contemplate and delve into the motives, emotions, and experiences that underlie their selected images.

We employed the photovoice approach to support our partners as they reflect on their own experiences and those of others, prompting them to create or capture images that convey their thoughts and analyze the images chosen by others.

Five partners first attended a 90-minute virtual photovoice workshop on Zoom, where they participated in interactive activities to gain/further their understanding of photography and how we can use photovoice.

Supported by the research team to present her experiences, one partner shared her journey of completing a photovoice activity (using a different prompt unrelated to this project) to provide reflections on the process and an example for others. At the end of the workshop, the partners were invited to develop a submission composed of a description and image response to the prompt “What would the ideal partnership/engagement of older adults be in the Collaborative?”. The recording of the workshop was available for those who did not attend. About a week later, 5 out of 9 partners shared their submissions and engaged in a 90-minute virtual discussion on Zoom regarding the impact, process, and responses to the photovoice prompt. One person was not able to submit due to health reasons but participated in the discussion by sharing reflections and experiences of viewing others’ submissions.

In addition to the recorded training workshop, partners were offered individual support through additional phone calls/Zoom.

### Focus group

The focus group aimed to encourage the partners to express their individual and collective experiences as older adult partners within the Collaborative. There were two sections to the focus group guide: (1) Overview and Impact of Participation within the Collaborative and (2) Partnership Challenges and Successes. The focus group, which lasted 90 min, took place online (via Zoom) and was attended by 6 out of 9 partners. See focus group questions below:

Section 1: Overview and impact of participation within the Collaborative

1. What impact has your participation in the Collaborative, as a partner, had on you?
2. How has your participation in the Collaborative as a partner influenced your self advocacy/general advocacy skills? (if at all)
3. What different perspectives do you bring to the Collaborative?

Section 2: Partnership challenges and successes.

1. What are some anticipated challenges for others who may be more marginalized due to social or health-related factors when participating or adopting the role of a partner? (Socioeconomic, gender/sex, race/ethnicity, religion/spirituality, physical and mental/cognitive capabilities)
2. Reciprocity is an important principle for partnership; what do you feel like you are “getting back” from your involvement in the Collaborative?
3. When you think about how this team works *together as a group* (a) what makes the initiative successful? (b) what gets in the way of success?

Most focus group questions were developed in collaboration with the partners whileothers were added by the research team in response to findings from the PPEET and other evaluation methods.

## Partnership during the analysis and knowledge mobilization phases

The student researcher and the Collaborative’s managing director conducted a thematic analysis and developed multiple outputs to engage the partners in a conversation about the preliminary findings and outputs. The aim of this was to help ensure that partner perspectives were appropriately interpreted and important themes were not missing. The preliminary findings were also used to identify and discuss potential next steps for the Collaborative to maintain and/or improve meaningful partnerships. We created and shared (1) an accessible video, presentation, and document, reviewing the methods and summarizing the findings and (2) an action report with clear next steps on how we could mobilize the study findings, as a starting point for discussion.

The research team and older adult partners met to reflect on the process as well as discuss the preliminary findings and ideas for sharing and putting the lessons learned into action.

Discussion questions included:

1. Did any of the quotes stand out to you as being extra powerful or perhaps not representative of the theme?
2. Do the themes represent your experience and/or the group discussions?
3. Is anything missing?

Additionally, two partners met with the Collaborative leaders and student researcher to discuss the project findings and, more specifically, how this work could contribute to the academic literature. The aim of these follow-up meetings was to decide on key messages and methods for mobilizing this knowledge within the academic and local community, including the current manuscript. As a result of the partners’ desire to see how outcomes of the evaluation inform the partnerships at the Collaborative, we created a report with key messages discussing the actions that were present and will be continued, that will be stopped, and will be started to promote meaningful partnerships. Examples include starting initiatives to acknowledge and celebrate partners, review communication to improve clarity, and continue welcoming diverse perspectives.

Considering that the findings have been presented in multiple formats (video, presentation, and accessible report), the project has become more accessible for sharing with larger audiences in the greater community of students, older adults, and other researchers with and without experience in partnership.

## Reflections on challenges, benefits, and limitations

There were benefits and limitations, identified by the research team and partners, associated with each method (see Table 1). Together, the methods complement one another by creating a study methodology that captures the nuances of each partner’s respective experiences, the process of partnership, and the evaluation of the partnership. Using multiple methods for partnership evaluation offers a range of benefits (see Table 1). Together, these methods created a comprehensive evaluation of partnership.The research team and partners determined that both quantitative and qualitative methods were needed for this participatory evaluation, and within each, multiple methods were desired to accommodate preferences and capture different elements of the partners’ perspective on their engagement.

**Table 1:** Benefits and limitations of each method used

Method	Benefits	Limitations
PPEET	Allowed partners to share their perspectives anonymously Includes demographic information Allows for descriptive statistical analysis Allows for comparisons across time and initiatives	Does not include much room for unrestricted feedback or expression of ideas Can be inaccessible to individuals with certain impairments (e.g., the requirement to complete the PPEET online added technological challenges) Does not allow identification of what is most important to each participant Limitations with analysis and generalizability arise with small sample sizes
Photovoice	Promoted individual and group reflection regarding the prompt Allowed for the creative expression of ideas Yields non-traditional and engaging ways to share findings	Some hesitated to participate due to perceived artistic demands of method and accessibility issues (e.g., visual acuity, mobility, etc.) Time and resource-consuming for the partners
Focus Group	Allows for reflection and unrestricted sharing Those who did not want to participate in photovoice had another option to share their ideas	Unlike photovoice, where partners had more time to think individually, focus group require immediate responses to questions

The success of the PPEET survey in meeting the objective of providing space to discuss negative aspects of partnership anonymously was clear. Specifically, more negative aspects of partnership were mentioned in the PPEET than other methods of evaluation. For example, the partners shared their desire for more accessible communication methods such as reviewing all communications to improve clarity and reduce the length of emails.

The outputs produced by the photovoice activity were diverse. Submissions included photos with symbolic creations (e.g., knitted material to reflect different perspectives woven together) and objects to answer the prompt, abstract art, and photographs that portrayed meaning more literally (e.g., image of holding hands to indicate support). During the discussion, some partners shared how it took them a lot of time and trial and error to embody their perspective with an artistic method as well as figure out what their perspective is. This displays how the objectives of using photovoice in order to allow for time to reflect on the prompt individually and portray perspective in a way that words may not allow were met. Ultimately, the method was successful in this regard.

Photovoice was more challenging for the partners than anticipated. Despite the photovoice training session, which aimed to simplify the process and provide guidance, some felt that they did not possess the necessary skills to enact this approach. Some also expressed hesitation, knowing that their submissions would be visible to other partners and identified as their own. This displays how participation and contributions may be influenced by partners’ desire to appear a certain way. Voicing their perspective that their submissions reflect them as individuals, the partners discussed the extensive time spent reflecting on the prompt, contemplating what they wished to express, how to portray it using photography and capturing an image they were pleased with. The research team provided the partners with approximately two weeks to complete their submission. This was done to accommodate schedules and avoid overwhelming the partners. However, some expressed that the longer time signaled greater expectations for the submissions and thus increased the stress of the activity. To mitigate these challenges, MA and SCC met with one of the partners for insight on how to make the photovoice process more accessible and to offer additional support. This meeting led to further conversations with the group of partners to provide rationale for the longer time frame and the photovoice activity expectations. Despite these challenges, partners expressed an appreciation for the opportunity to reflect on partnership as they listened to each other share their submissions and experiences. For many partners, photovoice was a creative channel for self-reflection and expression, and sharing their experiences in ways not confined to words alone. As such, photovoice might serve as a complementary or alternative strategy to tackle linguistic and cultural challenges inherent in many traditional forms of communication and research methods.

While photovoice presented challenges for some partners, the focus group was more accessible to the group. Its success was evident in the extensive reflections made by each partner as well as the diverse perspectives shared. It was notable how the partners built upon each others’points to indicate agreement or disagreement, leading to in depth discussion and allowing for collective reflections to share experiences regarding engagement within the Collaborative.

Another encountered challenge across the overall project was attendance. Although all partners were offered the opportunity to participate in all these methods and project stages, some were absent during some activities due to health issues or scheduling conflicts. It is important to be flexible and understanding when engaging with older adults to adapt your project to fluctuating health and competing priorities of partners [3, 31].

Ultimately, this project displays the numerous methods by which older adult partners can be engaged in the research process as partners and the valuable insights that they bring. Below we provide recommendations to guide meaningful partnership with older adults, thereby debunking the perception that the population is difficult to engage.

## Recommendations

We recognize that while including multiple methods within an evaluation of partnerships is beneficial, it may not be feasible for all evaluations due to time and resource constraints. We suggest that the partners and research team assess their goals and the partnership context to choose the most appropriate tools and methods. For example, if the evaluation entails discussing sensitive topics, an individual arts-based method such as photovoice may allow for self-expression in nonverbal and creative ways, time to reflect, and the opportunity to decide what to share in a private setting (if the photovoice activity is completed individually and on partners’ own time). While photovoice provides multiple benefits when discussing sensitive topics, it is worth noting that partners mentioned how they felt that their submissions represented them, which made some of them hesitant to share or conscious of their contributions. Thus, in such situations, using photovoice but analyzing the submissions or not sharing them widely is recommended [32]. It is important to discuss with the partners their level of comfort with sharing submissions. This discussion is crucial in encouraging more people to gain confidence and participate in the activity if privacy was their concern. Partner population and capabilities should also be considered when choosing tools and methods. For example, if the partners are not comfortable expressing their ideas visually and prefer traditional methods, accommodations should be made. Conversely, if partners have language barriers, using photovoice may be a better option than a focus group. As well, opportunities for sharing their insights one-on-one with a member of the research team can also be offered if they do not feel comfortable sharing in a group format.

## Conclusion

In summary, we share the experience of implementing a participatory evaluation of older adult partners’ engagement in a research Collaborative. The emphasis on co-planning and using both standardized tools and qualitative approaches underscores the comprehensive approach that was taken to assessing the partnerships within the Collaborative. We hope this case example offers helpful guidance to other groups as they think through the different approaches to partnership and its evaluation. We encourage researchers to consider if or how it may be beneficial to: (1) include partnership in every phase of the evaluation process, (2) Use multiple methods to capture experiences in different ways, (3) Provide individual and customizable support to partners to mitigate barriers due to background, health status, and resources.

## Study limitations

While we offer practical guidance on participatory evaluations, we want to emphasize that such guidance was based on the experience of our partners; other research teams should consider the appropriateness of these methods for their evaluation aims and alignment with the skills and preference of their partners when considering adopting these methods. It is important to note that a benefit of participatory approaches is that they are flexible to group needs and study objectives. Ultimatly, project objectives and partner preference should be the main drivers for evaluation approaches.

### Electronic supplementary material

Below is the link to the electronic supplementary material.

Supplementary Material 1

## Data Availability

No datasets were generated or analysed during the current study.
